# Impaired extraction of speech rhythm from temporal modulation patterns in speech in developmental dyslexia

**DOI:** 10.3389/fnhum.2014.00096

**Published:** 2014-02-24

**Authors:** Victoria Leong, Usha Goswami

**Affiliations:** Department of Psychology, Centre for Neuroscience in Education, University of CambridgeCambridge, UK

**Keywords:** amplitude modulation, envelope, speech rhythm, dyslexia, oscillations

## Abstract

Dyslexia is associated with impaired neural representation of the sound structure of words (phonology). The “phonological deficit” in dyslexia may arise in part from impaired speech rhythm perception, thought to depend on neural oscillatory phase-locking to slow amplitude modulation (AM) patterns in the speech envelope. Speech contains AM patterns at multiple temporal rates, and these different AM rates are associated with phonological units of different grain sizes, e.g., related to stress, syllables or phonemes. Here, we assess the ability of adults with dyslexia to use speech AMs to identify rhythm patterns (RPs). We study 3 important temporal rates: “Stress” (~2 Hz), “Syllable” (~4 Hz) and “Sub-beat” (reduced syllables, ~14 Hz). 21 dyslexics and 21 controls listened to nursery rhyme sentences that had been tone-vocoded using either *single* AM rates from the speech envelope (Stress only, Syllable only, Sub-beat only) or *pairs* of AM rates (Stress + Syllable, Syllable + Sub-beat). They were asked to use the acoustic rhythm of the stimulus to identity the original nursery rhyme sentence. The data showed that dyslexics were significantly poorer at detecting rhythm compared to controls when they had to utilize multi-rate temporal information from *pairs* of AMs (Stress + Syllable or Syllable + Sub-beat). These data suggest that dyslexia is associated with a reduced ability to utilize AMs <20 Hz for rhythm recognition. This perceptual deficit in utilizing AM patterns in speech could be underpinned by less efficient neuronal phase alignment and cross-frequency neuronal oscillatory synchronization in dyslexia. Dyslexics' perceptual difficulties in capturing the full spectro-temporal complexity of speech over multiple timescales could contribute to the development of impaired phonological representations for words, the cognitive hallmark of dyslexia across languages.

## Introduction

### Speech rhythm and phonological awareness in dyslexia

Dyslexia is characterized across languages by difficulties in phonological processing (e.g., Snowling, 2000; Ziegler and Goswami, 2005). Phonological processing encompasses the encoding and representation of speech at a range of grain sizes, both segmental (i.e., phoneme) and supra-segmental (e.g., rime, syllable and stress). As simple decoding (word reading) requires the acquisition of phonology-orthography correspondences at different grain sizes (segmental for alphabetic languages, syllabic for some character-based scripts), this cognitive “phonological deficit” affects reading acquisition in dyslexia across languages. While an impairment in segmental processing in dyslexia has long been noted (e.g., Tallal and Piercy, 1974; Snowling, 1981), supra-segmental sensitivity has only recently been a focus of study, and then mainly in English (e.g., Wood and Terrell, 1998; Goswami et al., 2002, 2010). This is surprising, as children's phonological sensitivity to supra-segmental features of speech develops early in all languages, well before the onset of formal literacy instruction. Indeed, EEG studies reveal sensitivity to the dominant stress patterns in the native language within the first months of life (Friederici et al., 2007; Ragó et al., 2014).

For English-learning infants, this early sensitivity toward dominant syllable stress patterns such as the “Strong-weak” (S-w) trochaic motif has been shown to be important for word learning (Jusczyk et al., 1993; Echols et al., 1997). By the age of 7.5 months, English-learning infants are capable of using the trochaic stress pattern as a template for segmenting words from continuous speech (Jusczyk et al., 1999). During early childhood, pre-literate children across languages already exhibit an awareness for rime and syllable units in speech. Pre-readers are able to identify pairs of words that rhyme (e.g., “mat” rhymes with “hat” but not with “cut”), and to clap out the number of constituent syllables in a word (Bradley and Bryant, 1983; Treiman and Zukowski, 1991; Ziegler and Goswami, 2005). In fact, children's phonological awareness of rhyme, syllables and stress predicts their later success in learning to read (Bradley and Bryant, 1983; de Bree et al., 2006; Whalley and Hansen, 2006).

Sensitivity to supra-segmental features of speech, particularly speech rhythm and syllable stress, also appear to be impaired in children and adults with developmental dyslexia (e.g., Wood and Terrell, 1998; Kitzen, 2001; Goswami et al., 2010; Holliman et al., 2010, 2012; Leong et al., 2011; Mundy and Carroll, 2012). Acoustically, prosodic rhythm and stress in the speech signal are cued by a combination of amplitude, duration and frequency changes (Hirst, 2006). The amplitude-based cues to rhythm are contained within the slow-varying “amplitude envelope” of speech (Plomp, 1983; Howell, 1984, 1988a,b; Greenberg et al., 2003; Tilsen and Johnson, 2008; Leong, 2012; Tilsen and Arvaniti, 2013). These slowly-varying amplitude patterns also cue the location of the rhythmic “perceptual (P)-center” or *moment of occurrence* of a sound (Allen, 1972; Morton et al., 1976; Scott, 1993, 1998; Villing, 2010). The P-center forms the basis for the deliberate rhythmic timing of speech and for synchronization of speech between speakers (Cummins and Port, 1998; Cummins, 2003). The P-center is related perceptually to a particular rhythmic marker within the speech amplitude envelope: the envelope onset rise time. Perceptual sensitivity to rise time is impaired in children and adults with dyslexia in a range of languages (Goswami et al., 2002; Hämäläinen et al., 2005, 2009; Surányi et al., 2009; Poelmans et al., 2011; Goswami et al., 2011a; see Goswami, 2011, for a recent summary). The rise time or “attack” time of a sound refers to the rate at which its amplitude increases during its initial onset, and is closely related to its P-center and rhythmic “beat strength.” For example, a trumpet note with a fast rise time and early P-center will typically be perceived as having a stronger beat than a bowed violin note with a slower rise time and later P-center (Gordon, 1987). In speech, envelope onset rise times distinguish between stressed and unstressed syllables (Leong et al., 2011; Goswami and Leong, 2013), and provide phonetic cues to voice onset time and manner of articulation, for example aiding in phonetic distinctions such as between /b/ and /w/ (Goswami et al., 2011b). Dyslexics' difficulties in perceiving amplitude envelope rise times across languages has led to the theoretical suggestion that a deficit in neural rhythmic entrainment to amplitude modulation (AM) patterns in speech could underlie the phonological deficit in developmental dyslexia (Goswami, 2011; “temporal sampling theory”).

### Neuronal oscillatory entrainment in dyslexia

The speech amplitude envelope contains a spectrum of AM at different temporal rates, with certain key rates of AM associated with characteristic timescales of speech information. For example, the envelope is dominated by modulations that occur at around 3–5 Hz, corresponding to the average duration of the syllable (Greenberg et al., 2003; Greenberg, 2006). AMs at a slower rate of ~2 Hz are associated with inter-stress intervals in speech, which have an average duration of 493 ms (Dauer, 1983). Toward the other end of the modulation spectrum, faster modulations immediately above the ‘classic’ syllable rate of 3–5 Hz correspond to more quickly-uttered unstressed syllables (~10 Hz, Greenberg et al., 2003). Faster modulations up to 50 Hz are thought to provide phonemic cues to manner of articulation, voicing, and vowel identity (Rosen, 1992). Although the amplitude envelope has been the focus of many speech intelligibility studies (e.g., Drullman et al., 1994a,b; Shannon et al., 1995), the spectral fine structure also makes an important contribution to speech intelligibility, particularly under adverse listening conditions (Qin and Oxenham, 2003; Xu et al., 2005; Obleser et al., 2012).

Recently, Poeppel and colleagues have proposed a neural account of speech processing based on multi-time resolution of the modulation patterns in the speech envelope (multi-time resolution models, e.g., Poeppel, 2003; Giraud and Poeppel, 2012). In multi-time resolution models, the brain is thought to track speech information at different timescales using neuronal oscillations at different frequencies. These neuronal oscillations *entrain* (“phase-lock”) to speech modulation patterns on equivalent timescales, so that peaks and troughs in oscillatory activity align with peaks and troughs in modulations in the signal. According to Giraud and Poeppel (2012), neuronal oscillatory activity in the Theta band (3–7 Hz) tracks syllable patterns in speech, while slower oscillatory activity in the Delta band (1–3) Hz tracks phrasal and intonational patterns, such as stress intervals. Fast oscillatory activity in the Gamma band (25–80 Hz) is thought to track quickly-varying phonetic information, such as formant transitions and voice-onset times, which have timescales in the order of tens of milliseconds. This convergence between characteristic timescales in speech and the dominant neuronal oscillatory bands in auditory cortex has been used to argue that oscillatory entrainment (“phase locking”) may be an important neural mechanism for parsing the speech signal into appropriately-sized linguistic units for further lexical processing (Ghitza and Greenberg, 2009; Schroeder and Lakatos, 2009; Giraud and Poeppel, 2012; Zion Golumbic et al., 2012).

In line with dyslexics' difficulties in rise time perception, which are particularly evident for slower rise times (Richardson et al., 2004; Stefanics et al., 2011). Goswami (2011) proposed a “temporal sampling” framework to explain why the development of accurate phonological representation of speech is impaired across languages in developmental dyslexia. The temporal sampling framework proposed that impaired phonological representation in dyslexia could arise in part from impaired oscillatory entrainment to *slow* AMs (<10 Hz) that carry stress and syllable patterning in speech (i.e., involving delta and theta oscillations, see Goswami, 2011; Power et al., 2012, 2013; Soltész et al., 2013). As neuronal oscillations in the cortex exhibit hierarchical nesting across slow and fast timescales (e.g., theta-gamma phase-amplitude coupling; Lakatos et al., 2005), an impairment in slow oscillatory activity (e.g., delta, stressed syllable rate; theta, syllable rate) could also have consequences for speech encoding at faster timescales, such as the Gamma or other phonetic rate timescales. Indeed, recent studies using non-speech stimuli have indicated that the hemispheric lateralization of Gamma-rate oscillations (~30 Hz) may be altered in dyslexia (Lehongre et al., 2011, 2013).

### AM perception in dyslexia

Consistent with Goswami's (2011) proposal, several AM perception studies based on non-speech stimuli and psychoacoustic modulation thresholds indicate that dyslexics show poor AM sensitivity below 10 Hz (e.g., Lorenzi et al., 2000; Amitay et al., 2002; Rocheron et al., 2002; although note that Poelmans et al., 2012 observed no deficit at 4 Hz). Studies reporting on modulation thresholds for faster AM rates vary in whether they report dyslexic deficits. For example, while McAnally and Stein (1997), Witton et al. (1998), and Menell et al. (1999) all observed deficits in dyslexics' AM detection at ~20 Hz, Hämäläinen et al. (2009) failed to find a deficit at the same rate. Meanwhile, while no dyslexic deficit at 80 Hz was reported by (Hari et al., 1999), a study by Poelmans et al. (2012) found atypical laterality effects in EEG for 20 Hz AM speech-weighted noise, and a study by Lehongre et al. (2011) found atypical laterality effects in MEG for 35 Hz AM white noise. Similarly mixed results have been observed for dyslexics' perception of very slow “stress rate” AMs. While an early study by Witton et al. (1998) found that the perception of 2 Hz AMs was unimpaired in dyslexia, subsequent studies by Stuart et al. (2006) and Hämäläinen et al. (2012) have reported significant group differences in AM sensitivity at the 1 Hz and 2 Hz rates respectively. From the non-speech studies, it is currently unclear whether dyslexics have a *general* deficit in AM perception that affects all modulation rates, or whether their deficit is *specific* to the AM rates <10 Hz that are identified in temporal sampling theory (Goswami, 2011). It is also possible that a single auditory anomaly, impaired phonemic sampling in left auditory cortex, accounts for the impaired phonological processing found in dyslexia (Lehongre et al., 2011).

**Figure 1:**
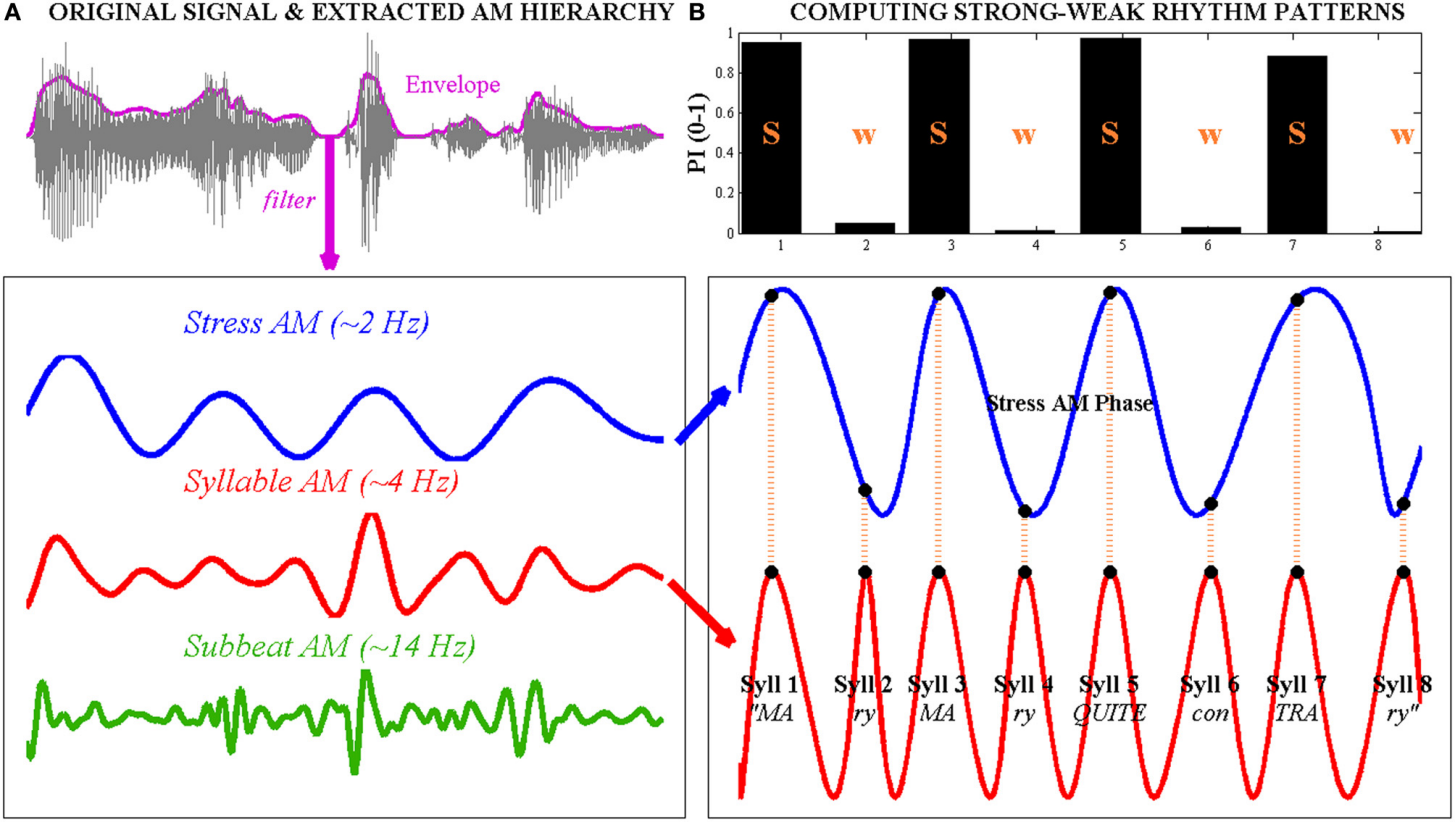
**Computation of strong-weak (s-w) syllable stress patterns using the phase-relationship between “Stress”- and “Syllable”-rate amplitude modulations (AMs) in the speech envelope, illustrated with the trochaic (s-w) nursery rhyme sentence “Mary Mary quite contrary.”** Left, **(A)** the original waveform of the speech signal is shown at the top, with the whole-band amplitude envelope superimposed as a bold line. The envelope is band-pass filtered at three different rates to produce a Stress AM (~2 Hz), a Syllable AM (~4 Hz) and a Sub-beat AM (~14 Hz) respectively. Right, **(B)** to compute the syllable stress pattern of the sentence, the oscillatory phase series of the Stress AM and the Syllable AM are extracted. Here, AM phase values are projected onto a cosine function for ease of visualization. Note that the 8 Syllable AM cycles correspond to the 8 spoken syllables in the sentence. The concurrent Stress AM phase at Syllable AM peaks (indicated with vertical dotted lines) is transformed into a prominence index (PI), shown in the bar graph at the top. Syllable AM peaks that occur near the oscillatory peak of the Stress AM achieve PI values of ~1, while Syllable AM peaks that occur near the oscillatory trough of the Stress AM achieve PI values of ~0. Here, syllables with a high PI (near 1) are considered “strong” while syllables with a low PI (near 0) are considered “weak.” Note that this Stress-Syllable AM phase relationship accurately reflects the trochaic syllable stress pattern of the sentence.

While AM studies are important for studying phase-locking, their implications for real-life speech perception are limited because the AM patterns used in these studies are artificial sinusoids and not real speech AMs. Real-speech AMs differ from artificial sinusoids in several important ways. First, unlike sinusoids, speech AMs are not perfectly periodically regular, but contain phase-advancements or delays that reduce their temporal predictability. Secondly, real-speech AMs differ in patterning at different acoustic frequencies. These temporal differences in modulation patterning across different “spectral channels” are crucial for speech intelligibility (e.g., Shannon et al., 1995). Finally, in real speech, AM patterns at all timescales (e.g., stress, syllable and phoneme) are *concurrently transmitted* to the listener, unlike artificial AM studies in which only one AM rate is presented at a time. During real-life speech processing, listeners probably extract speech information using *combinations* of AMs at different rates. For example, we have recently reported that listeners detect prosodic RPs by computing the *phase relationship* between two concurrent rates of speech AM: the “Stress” rate (~2 Hz) and the “Syllable” rate (~4 Hz, see Leong, 2012). This proposal is summarized in Figure 1. Dyslexics' ability to use such AM *combinations* in real speech has, to our knowledge, not been tested.

One obvious difficulty is that the complexity of the speech signal makes the extraction of specific features like cross-frequency AM phase alignment at pre-determined rates very difficult. Accordingly, studies using “vocoded” (envelope-only) real speech are useful. In vocoder studies, the speech signal is split into different frequency channels (e.g., typically 2, 4, 8 or 16 channels), the envelopes from each channel are used to modulate noise or tone carriers, and are then recombined. The resulting speech sounds like a harsh whisper, and is initially difficult to recognize. Speech vocoder studies with dyslexic children consistently suggest that their ability to use envelope cues for speech perception is impaired (e.g., Lorenzi et al., 2000; Johnson et al., 2011; Nittrouer and Lowenstein, 2013). For example, Lorenzi et al. (2000) used 4-channel noise-vocoded VCV syllables (e.g., /aCa/) as stimuli, and found that both typically-developing and dyslexic 11-year-old children performed more poorly than adults when using envelope cues (<500 Hz) for speech intelligibility. However, while the speech recognition performance of control children improved significantly over the course of five training sessions during the experiment, the performance of dyslexic children did *not* improve with training. Johnson et al. (2011) and Nittrouer and Lowenstein (2013) found more direct evidence for impaired speech envelope perception in dyslexia. In their study using 4- and 8-channel semantically-unpredictable noise-vocoded monosyllabic sentences (e.g., “dumb shoes will sing”), Johnson et al. (2011) found that 10–11 year-old children with reading difficulties showed significantly poorer word recognition of vocoded speech than control children, for both 4- and 8-channel stimuli. Similarly, Nittrouer and Lowenstein (2013) used 4-channel noise-vocoded sentences and found that there were consistent differences in speech perception performance between typically-developing and dyslexic children, for both age groups tested (8–9 years and 10–11 years).

In each of these studies, the vocoded stimulus typically contained a very wide range of envelope AM rates rather than a single AM rate (e.g., the envelope was low-pass filtered under 500 Hz). Thus, a complication of these experiments is that a deficit in perceiving speech modulations at a *specific* rate (e.g., 4 Hz) would be masked if the dyslexic children were able to extract redundant speech information at other modulation rates (e.g., 20 Hz) to compensate for a slow AM deficit (see Drullman, 2006). Conversely, if a difference in performance is observed (as was the case in these studies), it is not clear whether this is caused by a general deficit in AM processing that affects all modulation rates, a specific deficit at certain AM rates (e.g., pertaining to stress, syllable or phoneme-rate information), or a deficit in combining AM information across different temporal rates. Therefore, to assess speech AM perception in dyslexia more closely, a combination of the two approaches (from AM studies and vocoding studies) is needed. Ideally, the stimuli should be created from the envelopes of real speech, but AMs at specific modulation rates (or combinations of modulation rates) should be systematically isolated from these real envelopes. Here, we present one such study.

### Experimental rationale and hypotheses

Given the prior literature on the relationship between rhythmic awareness and reading (e.g., Thomson et al., 2006; Thomson and Goswami, 2008; Goswami and Leong, 2013; Tierney and Kraus, 2013), we were specifically interested in assessing dyslexics' ability to use different AM rates in speech for *rhythm perception* (rather than speech intelligibility *per se*). Accordingly, we devised a rhythm perception task using rhythmic sentences (nursery rhymes) that had been tone-vocoded using different AM rates. For normal adult listeners, speech rhythm perception relies on sensitivity to the phase-relationship between 2 key AM rates (stress ~2 Hz and syllable ~4 Hz; Leong, 2012). Furthermore, in prior work on rhythmic entrainment, we have shown that children and adults with dyslexia show “tapping to the beat” impairments at 2 Hz (Thomson et al., 2006; Thomson and Goswami, 2008), while when tapping to speech rhythms adults with dyslexia show impairment at the syllable rate (~4 Hz; Leong and Goswami, 2014). Accordingly, here we presented dyslexic and control adult listeners with tone-vocoded (envelope-only) sentences that contained only a narrow range of AM rates under 20 Hz. In order that the modulation patterns in our stimuli would be realistically speech-like, these modulation bands did not contain only a single AM rate (i.e., a “4 Hz” sinusoid). Rather each AM band contained a narrow range of AM rates centered around a target rate (e.g., 2.3–7 Hz, centered around 4 Hz), each of which we refer to in shorthand by the center rate (e.g., here as “~4 Hz” or “Syllable-rate AMs”).

Our dependent variable was the accuracy of speech rhythm perception. We created stimuli that contained modulations from either a single narrow AM band (i.e., Stress only ~2 Hz, Syllable only ~4 Hz, Sub-beat only ~14 Hz), or from *paired combinations* of AM bands (Stress + Syllable and Syllable + Sub-beat). On the basis of the temporal sampling framework (Goswami, 2011), we predicted no dyslexic impairment at the sub-beat band rate of ~14 Hz (included as a control frequency band), but significant impairment at both rates <10 Hz (Syllable and Stress rates). On the basis of our prior data on rhythmic entrainment to speech rhythms (Leong and Goswami, 2014), we also predicted that dyslexics would have difficulty in *combining* speech information across different temporal modulation rates. As Leong's modeling work (Leong, 2012) has shown that rhythm perception depends critically on the Stress + Syllable AM combination, it may be that particular dyslexic difficulty is found for this combination.

Note that in this experiment we used the ‘Sub-beat’ rate (~14 Hz) as a control AM band, not the “phoneme rate” (~30 Hz) that is the theoretical focus of AM work by Lehongre et al. (2011, 2013). Our decision was motivated by the classic psychophysical studies of Drullman et al. (1994a,b). These studies indicated that AM rates up to 16 Hz are the most important for speech intelligibility, and that the inclusion of faster AM rates *above 16 Hz* result in little improvement to intelligibility. Furthermore, in a rhythmic context, we noticed that unstressed syllables are often compressed to a “sub-beat” length in order to fit within the standard “beat” length of one ordinary syllable. For example, in the nursery rhyme sentence “Humpty Dumpty sat on the wall,” the syllables “sat” and “on” are compressed together, or reduced, to fit the space of one regular syllable like “Hum.” Consequently, the overall trochaic rhythm of the sentence is not disrupted. Thus, the “Sub-beat” rate (~14 Hz) is likely to correspond to speech modulations that are important for intelligibility, but which contribute little toward the overall rhythmic patterning of “Strong” and “weak” beats in a sentence, making this an ideal control modulation band. As the cited “phoneme” rate (~30 Hz) commonly refers to the timescale of formant transition patterns in speech (e.g., Giraud and Poeppel, 2012), we plan to examine this rate in the context of frequency modulation (FM) perception in future studies.

## Methods

### Participants

Twenty-one adults (9 M, 12 F) with developmental dyslexia and 26 control adults (7 M, 19 F) participated in the study. All dyslexic participants had received a formal diagnosis of developmental dyslexia and also showed significant reading and phonological deficits according to our own test battery. All participants had no other diagnosed auditory or learning difficulties, spoke English as a first language, and were aged under 40 years. As shown in Table 1, dyslexic and control participants were matched on IQ [2 subscales of the Wechsler Abbreviated Scale of Intelligence (WASI), Wechsler, 1999: A non-verbal subscale (Block Design) and a verbal subscale (Vocabulary)]. However, there was a significant age difference between dyslexic and control groups, where controls were slightly older on average [dyslexic mean age = 22.9 years; control mean age = 25.5 years; *F*_(1, 45)_ = 5.66, *p* < 0.05]. To account for this age difference, all our subsequent statistical analyses include age as a covariate. As this statistical solution is impartial, we felt that it would be preferable to manually excluding certain participants on the basis of their age, which would entail subjectivity as to how many and which participants to exclude.

**Table 1:** **Group performance on standardized ability, literacy and phonological tests**.

**Task**	**Dyslexic**	**Controls**	***F*_(1, 45)_**
Age	22.9	25.5	5.66^*^
(*SE*)	(0.6)	(0.8)	
IQ	129.6	129.8	0.01
(*SE*)	(1.0)	(1.5)	
- Non-Verbal IQ T score	70.6	70.7	0.01
	(0.7)	(0.8)	
- Verbal IQ T score	62.0	62.0	0.00
	(1.0)	(1.5)	
Auditory STM score (out of 16)	10.3	13.0	22.91^***^
(*SE*)	(0.4)	(0.4)	
Reading standard score	110.8	115.8	8.81^**^
(*SE*)	(1.4)	(1.0)	
Spelling standard score	104.7	117.0	43.68^***^
(*SE*)	(1.5)	(1.2)	
Phonology score (out of 30)	26.1	28.5	22.13^***^
(*SE*)	(0.4)	(0.3)	

* p < 0.05;

** p < 0.01;

*** p < 0.001.

Consistent with their diagnosis, dyslexics performed significantly more poorly than controls in standardized tests for literacy [Wide Range Achievement Test (WRAT-III), Reading and Spelling scales, Wilkinson, 1993] and phonological awareness (Phonological Assessment Battery (PhAB), Spoonerisms task, Fredrickson et al., 1997; Weschler Adult Intelligence Scale-Revised (WAIS-R) forward digit span subtest, Wechsler, 1981). Thus, despite the relatively high IQ of both groups (reflecting the fact that these were high-performing students at a world-class university), dyslexic participants still lagged behind their peers in their reading, spelling and phonological awareness skills. Both control and dyslexic participants also took part in other studies on rhythm perception and production (see also Leong and Goswami, 2014). Ethical approval for the study was obtained from the Cambridge Psychology Research Ethics Committee, and all participants were given a modest payment for taking part in the experiments.

### Materials

In line with our focus on rhythm, children's nursery rhymes were used as stimuli because these are a form of naturally-occurring, rhythmically-rich speech material, whose rhythm patterns (RPs) should be familiar to and easily identified by listeners. Four duple-meter nursery rhymes were used for the experiment, taking the first line of each nursery rhyme (8 syllables). The sentences fell into either of two RPs, as shown in Table 2. Two sentences had a “S-w” or trochaic pattern. These were “MA-ry MA-ry QUITE con-TRA-ry” and “SIM-ple SI-mon MET a PIE-man” (stressed syllables in CAPS). The other two sentences had a “w-S” or iambic pattern. These were “as I was GO-ing TO st IVES” and “the QUEEN of HEARTS she MADE some TARTS.” We chose to use trochaic and iambic patterns because these are the dominant prosodic motifs found in children's nursery rhymes (Gueron, 1974), and were easily understood by our participants. A total of 4 sentences (2 per RP) were used to encourage participants to attend to the global “S-w” or “w-S” rhythm patterning that was common between the 2 exemplars of each pattern. Using two exemplars also prevented reliance on minor non-rhythmic variations (e.g., total stimulus length) to perform the task. We did not use more than 4 sentences as this would have unnecessarily increased the difficulty of the task (which was already high in difficulty). Each sentence was ~2 s in length (Mary: 2.01 s; Simon: 2.12 s; St Ives: 2.37 s; Queen: 2.31 s). The nursery rhymes were spoken by a female native speaker of British English who was articulating in time to a 4 Hz (syllable rate) metronome beat. The speaker was instructed to produce the RP of each nursery rhyme as clearly as possible. Utterances were digitally recorded using a TASCAM digital recorder (44.1 kHz, 24-bit), and the metronome was not audible in the final recording.

**Table 2:** **List of nursery rhyme sentences and their rhythm pattern**.

**Rhythm pattern *(S, Strong; w, weak)***	**Nursery rhyme sentence *(CAPS, Strong syllable)***
**S** w **S** w **S** w **S** w **	“MA-ry MA-ry QUITE con-TRA-ry”
*(trochaic)*	“SIM-ple SI-mon MET a PIE-man”
w **S** w **S** w **S** w **S**	“as I was GO-ing TO st IVES”
*(iambic)*	“the QUEEN of HEARTS she MADE some TARTS”

### Rhythm perception task

In each trial, participants heard one of four tone-vocoded nursery rhyme sentences. They were asked to indicate the target sentence (one of four) by selecting an appropriate response button. Participants were told to base their judgment on the *RP* of the stimulus. Given that the vocoded sentences had a clear rhythm but were unintelligible (see Section Signal Processing Steps for Tone Vocoding), we did not expect participants' sentence identification to exceed 50% in accuracy (i.e., we expected accurate discrimination *between* trochaic vs iambic sentences, but not *within* 2 trochaic or iambic sentences). All participants were first given 20 practice trials, during which they heard the four sentences as originally spoken, without any vocoding. This enabled participants to learn the RP of each sentence, and to become familiar with the response button mapping. Subsequently, participants performed the task with tone-vocoded stimuli only. The tone-vocoded stimuli retained the temporal pattern of each nursery rhyme sentence, but were completely unintelligible. Cartoon icons representing the four response options were displayed on the computer screen throughout the experiment to help to reduce the memory load of the task. Auditory stimuli were presented diotically using Sennheiser HD580 headphones at 70 dB SPL. The experimental task was programmed in Presentation and delivered using a Lenovo ThinkPad Edge laptop.

#### Signal processing steps for tone vocoding

AM bands were extracted from the amplitude envelope of the speech signal of each nursery rhyme sentence using two different methods. In the first method, the amplitude envelope was extracted using the Hilbert transform. This Hilbert envelope was then passed through a modulation filterbank (MFB) of band-pass filters, which effectively isolated speech AMs corresponding to the (1) “Stress” rate (0.8–2.3 Hz), (2) “Syllable” rate (2.3–7 Hz), and (3) “Sub-beat” (7–20 Hz) rate. Please see Stone and Moore (2003) for details of the spectral filterbank design, which was adapted to be used as a MFB here. It is possible that artificial modulations may be introduced into the stimuli by the MFB method, since band-pass filters can introduce modulations near the center-frequency of the filter, through “ringing.” Therefore, a second AM-hierarchy extraction method was also used. This was Probabilistic Amplitude Demodulation (PAD; Turner and Sahani, 2011), and did not involve the Hilbert transform or filtering. Rather, the PAD method estimates the signal envelope using a model-based approach in which the signal is assumed to comprise the product of a positive slow envelope and a fast carrier. Bayesian statistical inference is used to invert the model, thereby identifying the envelope which best matches the data and the *a priori* assumptions (i.e., a positive-valued envelope whose mean is constant over time). This envelope extraction protocol can be run recursively at different timescales, yielding AMs at the same modulation rates as those derived from MFB filtering (Turner and Sahani, 2007; Turner, 2010). All participants heard both MFB-derived and PAD-derived vocoded stimuli in the same experiment. It was reasoned that if participants produced the same pattern of results with two methods of AM extraction that operate using very different sets of principles, the observed effects were likely to have arisen from real features in speech rather than filtering artifacts.

**Figure 2:**
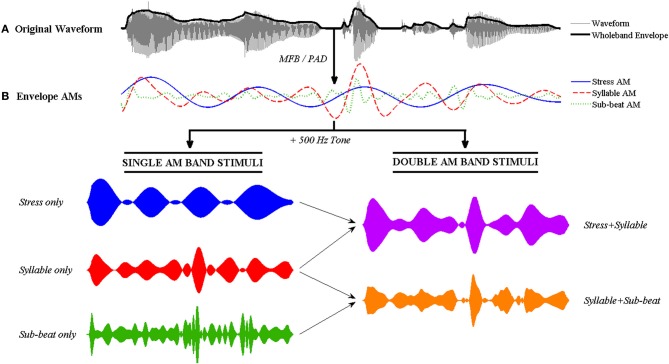
**Illustration of the signal processing steps involved in tone-vocoding for the nursery rhyme sentence “Mary Mary quite contrary.” (A)** The original speech signal with its wholeband amplitude envelope overlaid in bold. **(B)** The Stress AM, Syllable AM and Sub-beat AMs are extracted from the envelope using either the MFB or PAD method. Single and double AM band vocoded stimuli are then generated by combining the AMs with a 500 Hz sine tone. To generate single AM band stimuli (bottom left), each single AM band is multiplied individually with the sine tone. To generate double band AM stimuli (bottom right), the two AMs are first combined via addition (MFB) or multiplication (PAD) before multiplication with the sine tone. The resulting double band vocoded stimulus contains temporal patterning at two main rates (i.e., second-order modulation).

The MFB- and PAD-derived AMs were used to modulate a 500 Hz sine-tone carrier in a single-channel vocoder. A multi-channel vocoder was not used to ensure that the sentences would be completely unintelligible. As the dependent variable in the experiment was how well participants could identify each sentence on the basis of its AM RP, all other cues to sentence identity need to be removed. Therefore, the phonetic fine structure of the signal was intentionally discarded. In addition, the AMs derived from the amplitude envelope were used to modulate the sine-tone carrier, rather than being combined back with the fine structure of the signal. To create single-AM band stimuli (e.g., Stress only), the appropriate AM band was extracted and combined with the 500 Hz sine-tone carrier. A 30 ms-ramped pedestal at channel RMS power was added prior to combining with the carrier. To create double-AM band stimuli (e.g., Stress + Syllable), the two AM bands were first combined via addition (for MFB) or multiplication (for PAD) before combining with the carrier. All stimuli were equalized to 70 dB. These signal processing steps are illustrated in Figure 2.

**Figure 3:**
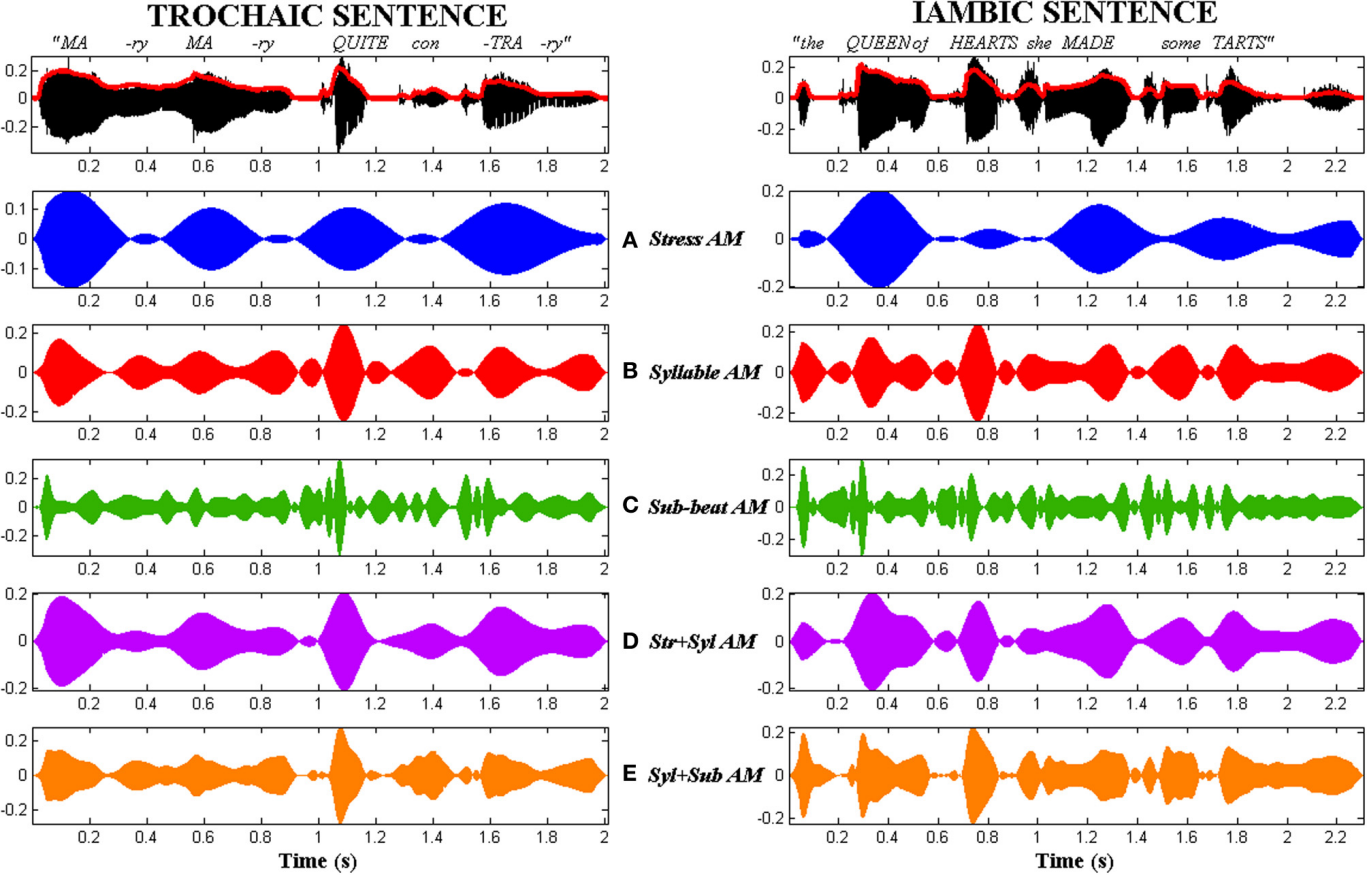
**Comparison of the 5 types of AM tone-vocoded stimuli produced for trochaic (S-w) and iambic (w-S) nursery rhyme sentences.** Stimuli corresponding to the trochaic sentence “Mary Mary” are shown in the left column. Stimuli corresponding to the iambic sentence “the Queen of Hearts” are shown in the right column. Top row: Original acoustic waveform of each sentence in black, with whole-band amplitude envelope overlaid in red. Rows **(A–E)** Stress AM, Syllable AM, Sub-beat AM, Stress + Syllable AM and Syllable + Sub-beat AM stimuli respectively.

The resulting tone-vocoded sentences had clear temporal patterns ranging from “Morse-code” to flutter, but were otherwise completely unintelligible (See Audios 1–5 in Supplementary Material). Figure 3 illustrates the different types of AM-vocoded stimuli used in the experiment, contrasting trochaic (“Mary Mary”) and iambic (“the Queen of Hearts”) sentences.

#### Design

As explained in Section Experimental Rationale and Hypotheses, five different AM bands or band combinations were used for vocoding. This generated 3 types of single AM band stimuli (Stress only; Syllable only; Sub-beat only) and 2 types of paired AM band stimuli (Stress + Syllable; Syllable + Sub-beat). For each AM combination, each of the 4 nursery rhyme sentences was presented 10 times (5 MFB and 5 PAD stimuli) in a fully randomized order, giving 40 trials per AM type and 200 trials in total for the entire experiment. Participants were scored in terms of their sentence identification accuracy for each AM type (Accuracy scores), and their ability to discriminate more generally between trochaic and iambic RPs (RP scores). We had previously found that control participants showed no difference in listening accuracy for MFB and PAD stimuli (Leong, 2012). In our preliminary analysis of the current data, we likewise found that there was no difference in performance for PAD as compared to MFB stimuli [*F*_(1, 44)_ = 2.74, *p* = 0.11]. Therefore, to simplify further analysis, the scores for the two types of stimuli in each condition were averaged into a single mean score for each participant.

## Results

### Sentence identification accuracy

**Figure 4:**
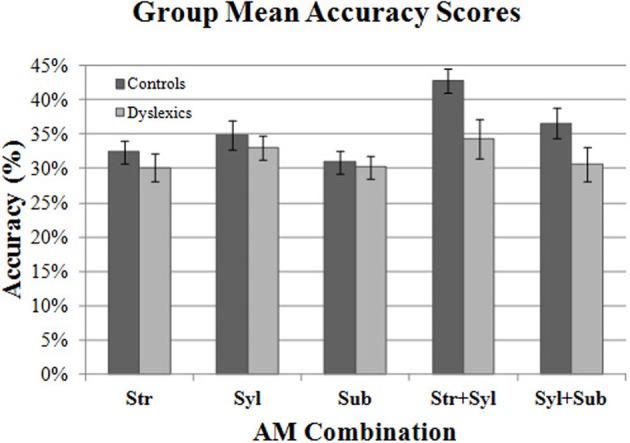
**Group mean Accuracy scores for each AM band and band combination.** Error bars indicate standard error.

Figure 4 shows the mean Accuracy scores achieved by the control and dyslexic groups for each AM type. To check for floor effects in performance (which could obscure group differences), we assessed whether participants' scores for each AM type were significantly above the level of chance (25%). Accordingly, separate one-sample *t*-tests were conducted for control and dyslexic groups against the test value of 0.25. As this necessitated 10 *t-tests* in total, Holm's sequential Bonferroni correction was applied to the *p*-value threshold for significance (Holm, 1979). Holm's sequential Bonferroni correction entails a smaller reduction in statistical power than the standard Bonferroni correction, and is a widely-used alternative for controlling for Type 1 family-wise error (Rice, 1989; Perneger, 1998). In the Holm-Bonferroni method, the threshold for significance is computed as 0.05/(10- [rank of uncorrected *p*-value] +1). Therefore, for the smallest (rank 1) *p*-value, the Holm Bonferroni-corrected threshold for significance was 0.05/(10 − 1 + 1) = 0.005, whereas for the largest (rank 10) *p*-value, the threshold for significance was 0.05/(10 − 10 + 1) = 0.05. The results of the *t*-tests indicated that both controls and dyslexics performed significantly above chance for all 5 AM types. Accordingly, we investigated whether there were group differences across the 5 AM types.

Two repeated measures ANCOVA analyses were conducted. In the first analysis, we compared group performance for the 3 *single* AM bands (Stress only, Syllable only, Sub-beat only). Single AM band (3 levels) was entered into the ANCOVA as the within-subjects factor, and Group (2 levels) was entered as the between subjects factor. Age was entered as a covariate factor. The results of the first ANCOVA showed *no* significant main effect of Group [*F*_(1, 44)_ = 0.14, *p* = 0.71], and no interaction between single AM band and Group [*F*_(2, 88)_ = 0.37, *p* = 0.69]. This suggests that controls and dyslexics were performing equally well in their use of single AM-band information for rhythm perception.

In the second RM ANCOVA analysis, we investigated group differences in the ability to combine information across more than one AM band. The second ANCOVA entered double-AM band (2 levels, Stress + Syllable, Syllable + Sub-beat) as the within-subjects factor, and Group (2 levels) as the between subjects factor. Age was again entered as a covariate factor. This second ANCOVA showed a significant main effect of Group [*F*_(1, 44)_ = 4.51, *p* < 0.05], but the interaction between AM band and Group did not approach significance [*F*_(1, 44)_ = 0.19, *p* = 0.66]. Therefore, our dyslexic participants were worse at combining AM information across different rates, as they were significantly less accurate than control participants. For combined AM bands, the dyslexic participants were significantly poorer at combining the Syllable-rate AM with other AMs at the Stress rate or the Sub-beat rate.

### Rhythm pattern discrimination

**Figure 5:**
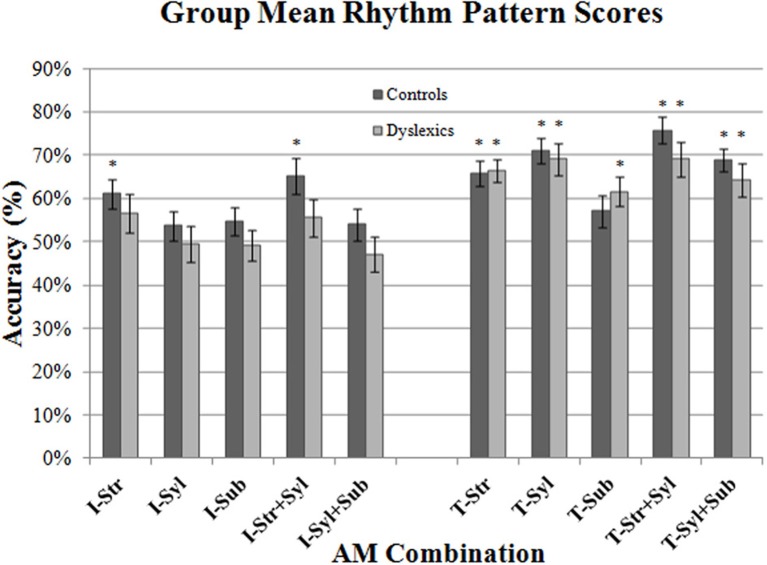
**Group mean Rhythm Pattern scores for each AM band and band combination, shown separately for iambic (“I”: Ives & Queen) and trochaic (“T”: Mary & Simon) sentences.** Error bars indicate standard error. (^*^) AM bands where performance was above chance (50%) for each group.

Next, we wanted to ascertain whether participants were able to use these speech AMs to discriminate between the two major RPs that characterized the 4 nursery rhyme sentences [i.e., trochaic (“S-w”) vs. iambic (“w-S”)]. Accordingly, we re-scored participants responses according to whether they had correctly identified the *RP* of each sentence as trochaic or iambic, disregarding whether they had identified the actual sentence correctly (i.e., for the stimulus sentence “Mary Mary,” responses of “Mary Mary” and “Simple Simon” were both scored as the correct RP, as both were trochaic responses). The resulting mean RP scores for iambic sentences (Ives, Queen) and trochaic sentences (Mary, Simon) are shown in Figure 5. To check for floor effects in performance (which could obscure group differences), we assessed whether participants' scores for each AM type were significantly above the level of chance (50%). Accordingly, separate one-sample *t*-tests were conducted for control and dyslexic groups against the test value of 0.5. As this necessitated 20 *t*-tests in total, Holm's sequential Bonferroni correction was applied to the *p*-value threshold for significance (Holm, 1979). For the smallest (rank 1) *p*-value, the Holm Bonferroni-corrected threshold for significance was 0.05/(20 − 1 + 1) = 0.0025, whereas for the largest (rank 10) *p*-value, the threshold for significance was 0.05/(20 − 20 + 1) = 0.05.

As shown in Figure 5 (*), controls and dyslexics always performed significantly above chance when making a binary discrimination of the rhythm of trochaic (T) sentences (with the exception of controls in the Sub-beat AM condition). By contrast, for iambic (I) sentences, dyslexics *never* performed above chance in binary rhythm discrimination, whereas controls performed significantly above chance when listening to Stress-only, and Stress + Syllable AM types. Given the presence of clear floor effects for binary rhythm discrimination of iambic sentences, we were unfortunately unable to draw further conclusions regarding group differences for these sentence types (as both controls and dyslexics were performing at chance in many conditions). However, both groups had performed significantly above chance for trochaic sentences when listening to Stress only AMs, Syllable only AMs, Stress + Syllable AMs and Syllable + Sub-beat AMs. According, we performed repeated measures ANCOVAs on these RP scores for trochaic sentences only.

In the first ANCOVA analysis, we compared group performance for the 2 single AM bands only, taking single AM band (2 levels) as the within-subjects factor, Group (2 levels) as the between subjects factor, and Age as the covariate. Consistent with the previous Accuracy analysis, there was *no* significant main effect of Group [*F*_(1, 44)_ = 0.16, *p* = 0.69], and no interaction between single AM band and Group [*F*_(1, 44)_ = 0.11, *p* = 0.75]. This suggests that controls and dyslexics did not differ in their ability to use Stress only and Syllable only AM band information to make trochaic-iambic distinctions. We then analyzed double-AM band performance in a similar fashion. This time double-AM band (2 levels, Stress + Syllable, Syllable + Sub-beat) was the within-subjects factor, Group (2 levels) was the between subjects factor, and Age was the covariate. Unlike the Accuracy analysis, the ANCOVA showed no significant main effect of Group [*F*_(1, 44)_ = 1.90, *p* = 0.17]. There was also no interaction between double-AM band and Group [*F*_(1, 44)_ = 0.17, *p* = 0.68]. Hence dyslexic participants appeared to recognize trochaic RPs based on pairs of AM as well as controls.

These results should be interpreted with caution, however. Firstly, only performance for trochaic sentences could be analyzed meaningfully (meaning that half the total dataset could not be analyzed). Secondly, the RP scores computed here reflect participants' rhythm discrimination *indirectly* rather than directly. The RP scores measure the *perceptual confusability* of sentences (i.e., how participants make guesses when they are unsure of the correct sentence identity). Perceptual confusability will depend in large part on the global RPs of the stimuli, but will also include other factors like total duration and perceptual grouping effects, as well as participants' own cognitive strategies. Nevertheless, the data show that perceptual confusability was maximal for trochaic sentences, for both groups.

### Correlations between AM perception, phonology, and literacy

By hypothesis, a perceptual deficit in using AM patterns to discriminate rhythmic sentences should be related to both phonological awareness and reading skills in our participants. Accordingly, we investigated the relationship between participants' sentence identification Accuracy for each AM band or combination, and their performance on memory, reading and phonological tasks. Table 3 shows the partial correlation matrix between accuracy of performance in the rhythm perception task (by AM type) and participants' memory, reading, and phonological ability, with age and IQ controlled. Correlations were performed with both groups combined, as well as separately. As shown in Table 3, there were several significant relationships between AM performance, literacy and phonology. Taking the group as a whole, the conceptually important Stress + Syllable speech AMs were significantly related to phonological awareness (*r* = 0.40, *p* < 0.01), as well as to auditory short-term memory (digit span, *r* = 0.35, *p* < 0.05). Performance with the Syllable + Sub-beat level was also significantly associated with spelling performance, which was not predicted (*r* = 0.32, *p* < 0.05). When considering the dyslexic group alone, the table shows that dyslexics' phonological awareness was significantly related to their sensitivity to Stress + Syllable speech AMs (*r* = 0.52, *p* < 0.01), while the relationship between Syllable AM performance and phonological awareness approached significance (*r* = 0.42, *p* = 0.074). Further, spelling skills were significantly related to Sub-beat AM sensitivity (*r* = 0.48, *p* < 0.05). Dyslexics also showed a significant relationship between their auditory short-term memory skills and their performance in the two combined AM conditions (*r* = 0.52, *p* < 0.05 for Stress + Syllable; *r* = 0.55, *p* < 0.05 for Syllable + Sub-beat). This may indicate that dyslexics' ability to use multiple patterns of temporal information to recognize speech rhythm in our experimental paradigm was constrained by their lower short-term memory capacity in comparison to controls. When considered as a group, controls showed no significant relationships between performance in the AM RP recognition task, phonology and reading, although there was a trend toward a correlation between Sub-beat AM sensitivity and spelling (*r* = 0.38, *p* = 0.07). Overall, therefore, the partial correlations show that the perceptual deficit in using AM patterns to detect speech rhythm was related to phonological awareness for the dyslexic participants only.

**Table 3:** **Pearson's r partial correlation values between accuracy of performance in rhythm perception (by AM type), and general ability, literacy and phonology measures**.

**Partial correlations controlling for Age and IQ**	**AM Combination**									
	**Stress only**		**Syllable only**		**Sub-beat only**		**Stress + Syllable**		**Syllable + Sub-beat**	
**AUDITORY STM**										
All	0.05		0.11		0.13		0.35^*^		0.17	
Con		−0.07		0.06		0.09		−0.09		−0.34
Dys		0.07		0.31		0.26		0.52^*^		0.55^*^
**READING**										
All	−0.14		0.02		0.21		0.13		0.21	
Con		−0.07		0.13		0.38^$^		−0.21		0.09
Dys		−0.32		0.03		0.14		0.17		0.18
**SPELLING**										
All	−0.17		0.12		0.25^^^		0.17		0.32^*^	
Con		−0.15		0.12		0.28		−0.09		0.11
Dys		−0.38		−0.06		0.48^*^		0.05		0.27
**PHONOLOGY**										
All	0.13		0.30^*^		0.18		0.40^**^		0.11	
Con		0.04		0.27		0.22		−0.12		−0.16
Dys		0.17		0.42^&^		0.21		0.52^*^		0.07

For each cell, correlations over both groups are shown on the top left, correlations for controls only are shown on the middle right, and correlations for dyslexics only are shown on the bottom right. Age and IQ are controlled in all the correlations.

* p < 0.05;

** p < 0.01;

$ p = 0.07;

& p = 0.074;

∧ p = 0.096.

## Discussion and conclusion

Here, we tested the hypothesis that perceptual difficulties in processing the AM patterns in speech that yield speech rhythm are associated with the development of impaired phonological representations for words by dyslexic individuals. The development of impaired phonological representations of speech is the cognitive hallmark of dyslexia across languages (Snowling, 2000; Ziegler and Goswami, 2005; Goswami, 2011). We tested the sensitivity of adults with dyslexia to AM patterning yielding speech rhythm for several different AM bands and band combinations below 20 Hz that are present within the amplitude envelope of speech. We found that dyslexic participants performed significantly more poorly than control adults when they were required to combine Syllable-rate AMs with AMs at other rates (Stress + Syllable or Syllable + Sub-beat).However, the dyslexic participants performed on par with controls when asked to utilize the temporal information at a single AM rate only (Stress only, Syllable only, or Sub-beat only). Accordingly, we conclude that dyslexics' difficulties with AM perception appear to occur across *more than one* speech timescale (particularly involving the Syllable rate). Moreover, as predicted by the temporal sampling framework, a perceptual deficit in utilizing AM patterns in speech is related to phonological development in dyslexia.

A deficit in Syllable-rate *combination* or *synchronization* with other rates would support the findings of Leong and Goswami (2014), in which the same group of adult dyslexics tested here showed differences in their *phase* of rhythmic entrainment at the Syllable rate in a rhythmic tapping task to nursery rhyme targets. A difference in Syllable *phase* of entrainment suggests that dyslexics have temporal differences in their processing of Syllable-rate information (e.g., they may perceive P-centers as occurring earlier in a speech sound as compared to controls). Here, participants with dyslexia were significantly poorer at recognizing the target nursery rhymes when they had to combine Syllable AM cues with prosodic stress AM cues (Stress + Syllable).

In fact, a circular-linear correlation analysis of the two datasets (Leong and Goswami, 2014 and the current study) revealed that there was a strong correlation between participants' Syllable AM phase of tapping in the entrainment task based on rhythmic tapping, and their sensitivity to Stress + Syllable AMs in the current task (*r* = 0.55, *p* < 0.01). An earlier Syllable AM phase of rhythmic tapping in Leong and Goswami (2014) was associated with poorer perception of Stress+Syllable AMs in the current study. No other AM band in the current study yielded significant correlations with tapping phase in the prior study. Others have argued that the perception and production of rhythm both rely on similar cognitive and neural mechanisms, such as the entrainment of neuronal oscillatory activity (Martin, 1972; Liberman and Mattingly, 1985; Kotz and Schwartze, 2010). In the current context, it is note-worthy that the common locus of dyslexic deficit across perception and production tasks involved the Syllable-rate of temporal processing.

Utilizing younger participants, Power et al. (2013) have shown in a rhythmic speech processing task that children with dyslexia also have a different preferred phase of entrainment in the *delta* band (2 Hz), both in response to auditory speech alone, and when speech information is audio-visual. The ‘temporal misalignment’ of both stress- and syllable-rate information in dyslexia found by Power et al. (2013) and the current study could explain why individuals with dyslexia develop phonological representations for words that are impaired (or specified differently) in comparison to those of unaffected individuals. If temporal processing of slower-rate information in speech is impaired, for example because oscillatory phase alignment is inaccurate, then this would affect the development of the entire mental lexicon of word forms, not simply of syllable-level and prosodic information. If syllable stress representation and syllabic parsing is different in dyslexia because of a perceptual deficit in utilizing AM patterns in speech, this would also affect phonetic-level information. Phonemes are perceived more accurately when they are in stressed syllables (Mehta and Cutler, 1988). Over the course of development, if dyslexic children consistently fail to capture rich, high-dimensional representations of the temporal patterns that occur on multiple timescales in speech (e.g., concurrently encoding Stress patterns, Syllable patterns and Phoneme patterns into an integrated representation of a word), this would yield the impoverished or atypical phonological representations that are developed by children with dyslexia across languages.

At first glance, our data appear to be inconsistent with the results of previous AM perception studies as summarized in the Introduction. These non-speech studies generally indicated that individuals with dyslexia had poorer AM perception at the 4 Hz rate (Syllable AM). Here, we find no differences in performance between controls and dyslexics when making rhythm judgments on the basis of the Syllable AM (4 Hz) only. However, it should be noted that the dependent variable being assessed in the current study is different from that of psychophysical AM studies. Whereas AM studies assess modulation detection *thresholds* based on just noticeable differences in modulation depth or rate (e.g., Lorenzi et al., 2000; Rocheron et al., 2002), here we assess nursery rhyme recognition using real-life speech AMs that contain strong (and likely supra-threshold) modulation patterns. As such, it is not surprising that no group differences were observed for our single AM rate stimuli. It is possible that significant group differences could have been observed at single AM rates if we had used sentences with weaker modulation patterns, such as whispered or mumbled speech. However, we *did* observe a significant difference in dyslexics' ability to *combine or integrate* speech modulation patterns across the Stress and Syllable rates, which is consistent with dyslexics' poorer speech perception performance in vocoder studies (e.g., Lorenzi et al., 2000; Johnson et al., 2011; Nittrouer and Lowenstein, 2013). This difference cannot be attributed to a general lack of attention or engagement by dyslexic participants, since they performed as well as controls with the single AM band stimuli. Rather, dyslexics appear to have a particular difficulty in making use of modulation information that is patterned at more than one timescale, here when Syllable-rate information has to be temporally synchronized with Stress-rate speech information or Sub-beat information. However, as we did not include paired AM combinations that did *not* involve the Syllable AM rate (e.g., Stress + Phoneme), we are not able to determine whether this difficulty is specific to Syllable AM combinations only, or whether it would also occur for other combinations of speech AMs.

It should also be observed that our participants found the rhythm judgment task very difficult. This high level of difficulty stemmed from the fact that the sentences were (deliberately) unintelligible, forcing our participants to rely solely on the acoustic modulations in the stimuli to perform rhythm judgments, without recourse to lexical factors. Consequently, accuracy scores for both controls and dyslexics (although significantly above chance) were relatively low (below 50%). In future studies, the issue of task difficulty may be ameliorated by using a tone-vocoder with more than 1 spectral channel (i.e., 3 or 4 channels), which would have the effect of increasing speech intelligibility. However, increasing the intelligibility of the stimuli would also introduce a new confound: participants would now be able to use their lexical knowledge to augment their perceptual judgments of speech rhythm. Nonetheless, this trade-off might produce stronger effects. Lexical “boot-strapping” effects could be reduced by using semantically unpredictable sentences (following Johnson et al., 2011).

According to the temporal sampling framework (Goswami, 2011), the combination impairment for Stress + Syllable rate AMs found here should affect speech perception even when listening to *clear (i.e., fully intelligible) speech*, which has strong modulation patterns that are above the threshold for detection. Interestingly, this was exactly what Lorenzi et al. (2000) found in their study. They reported that dyslexic children performed significantly more poorly than adults and control children even when listening to clear, unprocessed (not-vocoded) VCV syllables (these syllables will contain significant Syllable-rate modulation, but not Stress-rate modulation). This controversial result might possibly be explained by other factors like memory or attention, nonetheless data like these suggest that speech AM perception in dyslexia clearly requires more investigation. Current data suggest that individuals with dyslexia are less sensitive to small changes in modulation depth and rate, particularly around the syllable and stress rates in speech. Future studies should explore how dyslexics' difficulties with processing slow modulations affects their ability to integrate and synchronize slow-varying stress and syllable information with more quickly-varying phoneme-rate information in speech. These perceptual difficulties could be one source of the impaired or atypical phonological representations stored in the mental lexicon of word forms by dyslexic individuals.

Finally, we note that, given recent proposals by Poeppel and colleagues regarding neural oscillatory phase-locking to speech modulation patterns (e.g., Ghitza, 2011; Giraud and Poeppel, 2012), the perceptual difficulties that we observe here could be underpinned by impaired phase alignment and cross-frequency phase synchronization between different neuronal oscillatory rates. For example, dyslexics could have poorer neuronal oscillatory synchronization between theta oscillations (syllable rate) and delta (stress rate) or gamma (phoneme rate) oscillations in the cortex. Similarly, the neural interplay between theta (syllable rate) and alpha (8–13 Hz, similar to the sub-beat rate here) oscillations during speech comprehension might be atypical in dyslexia as well (Obleser and Weisz, 2012). To date, such *cross-frequency neural synchronization* has not been studied in dyslexia (although see Leong and Goswami, 2014, for an assessment of cross-frequency *AM* synchronization in dyslexics' speech). Such studies could be very informative in the quest to identify cross-linguistic perceptual and neural deficits underpinning cognitive markers such as impaired phonology in developmental dyslexia.

### Conflict of interest statement

The authors declare that the research was conducted in the absence of any commercial or financial relationships that could be construed as a potential conflict of interest.

## Supplementary material

Audio 1 **Stress-only AM (MFB, Trochaic)**.

Audio 2 **Syllable-only AM (MFB, Trochaic)**.

Audio 3 **Sub-beat only AM (MFB, Trochaic)**.

Audio 4 **Stress + Syllable AM (MFB, Trochaic)**.

Audio 5 **Syllable + Sub-beat AM (MFB, Trochaic)**.
